# Variation in the sacrum of phytosaurs: New evidence from a partial skeleton of *Machaeroprosopus mccauleyi*

**DOI:** 10.1111/joa.14007

**Published:** 2024-01-29

**Authors:** Caleb N. LePore, Matthew A. McLain

**Affiliations:** ^1^ Department of Earth and Biological Sciences Loma Linda University Loma Linda California USA; ^2^ Department of Biological and Physical Sciences The Master's University Santa Clarita California USA

**Keywords:** Chinle Formation, Petrified Forest National Park, phytosaur, postcranial anatomy, sacral region, Triassic archosauriforms

## Abstract

Phytosaurs are a group of Upper Triassic semi‐aquatic archosauriform reptiles. Their variable skull morphology forms the foundation of our understanding of their relationships and paleoecology, while only a few studies have focused on demonstrating the existence of postcranial variation. The numbers of vertebrae in the sacrum are thought to vary from two, the plesiomorphic condition for archosauriforms, to three, with the addition of a sacralized dorsal (i.e., dorsosacral) vertebra. In this study, we demonstrate the presence of a sacralized first caudal (i.e., caudosacral) vertebra in a sacrum belonging to *Machaeroprosopus mccauleyi*. We rule out taphonomic distortion or pathology as explanations for the inclusion of this element in the sacrum, suggesting instead that it occurred through modifications of the same developmental processes that likely produced dorsosacral vertebrae in phytosaurs. Additionally, we show that a dorsosacral vertebra is common in phytosaur specimens from the Chinle Formation and Dockum Group of the southwestern United States and suggest that it may be widespread among phytosaurs. The addition of sacral vertebrae potentially aided adaptation to larger body sizes or more terrestrial lifestyles in certain taxa.

## Graphical abstract

Phytosaurs, a group of Triassic semi‐aquatic archosauriform reptiles, are thought to vary in their numbers of sacral vertebrae from two, the plesiomorphic condition for archosauriforms, to three, with the addition of a sacralized dorsal (i.e., dorsosacral) vertebra. In this study, we demonstrate the presence of a sacralized first caudal (i.e., caudosacral) vertebra in a sacrum belonging to *Machaeroprosopus mccauleyi*. Additionally, we show that a dorsosacral vertebra is common in phytosaur specimens from the Chinle Formation and Dockum Group of the southwestern United States and suggest that it may be widespread among phytosaurs. The addition of sacral vertebrae potentially aided adaptation to larger body sizes or more terrestrial lifestyles in certain taxa.

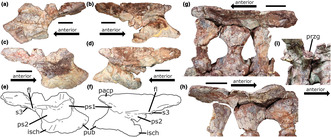

## 1 INTRODUCTION

Phytosaurs are an extinct lineage of non‐archosaurian archosauriform (Nesbitt, 2011) or pseudosuchian (Ezcurra, 2016) reptiles found as fossils in Upper Triassic continental deposits. They bear a strong superficial resemblance to extant crocodylians, being quadrupedal with short limbs and a long, mediolaterally compressed tail (Parrish, 1986). All known phytosaurs (save the Middle Triassic form *Diandongosuchus*; Stocker et al., 2017) possess elongated rostra and external nares positioned dorsally, just anterior to the orbits rather than at the terminal end of the rostrum. The rostrum may be narrower and gracile or wider and robust (Hunt, 1989), and may possess a crest, a feature which has been linked with sexual dimorphism (Zeigler et al., 2002). Additionally, phytosaur taxa display considerable variation in the morphology of their supratemporal fenestrae and squamosals (Ballew, 1989; Long & Murry, 1995). While most recent work on phytosaurs has focused on understanding their relationships and paleoecology based on skull anatomy (e.g., Jones & Butler, 2018a; Stocker, 2010), research on their postcranial skeleton has begun to shed light on variations in the postcranial anatomy of phytosaurs (Devore et al., 2020; Griffin et al., 2017; Grimes & McLain, 2019; Kimmig, 2013). With more comprehensive study, it may be possible to use such variation to clarify phytosaur relationships in areas where current phylogenies are unresolved (e.g., the most speciose sub‐clade, Leptosuchomorpha; [Jones & Butler, 2018a]). The number of vertebrae in the sacrum of phytosaurs is believed to vary between 2 and 3, with some individuals possessing a sacralized last dorsal vertebra (dorsosacral) in addition to the two primordial sacrals (Griffin et al., 2017).

In an effort to study sacral variation in phytosaurs, we examined the sacrum and ilia from a well‐preserved, partially complete, partially articulated skeleton of a phytosaur (PEFO 31219) from the Petrified Forest Member of the Chinle Formation within Petrified Forest National Park. We compared it with sacral vertebrae, ribs, and ilia from other phytosaurs collected in North American Upper Triassic deposits. We found that the sacrum of PEFO 31219 consists of at least three sacral vertebrae, which we interpret as the primordial first and second sacral vertebrae and a sacralized first caudal (caudosacral) vertebra.

Institutional Abbreviations: AMNH FARB, American Museum of Natural History Fossil Amphibians, Reptiles, and Birds collection, New York, New York; ISI, Indian Statistical Institute, Kolkata, India; MCCDM, Mesalands Community College Dinosaur Museum, Tucumcari, New Mexico; MNA, Museum of Northern Arizona, Flagstaff, Arizona; NMMNH, New Mexico Museum of Natural History and Science, Albuquerque, New Mexico; PPHM, Panhandle Plains Historical Museum; PEFO, Petrified Forest National Park, Arizona; TTU, Museum of Texas Tech University, Lubbock, Texas; UCMP, University of California Museum of Paleontology, Berkeley, California; UMMP, University of Michigan Museum of Paleontology, Ann Arbor, Michigan; UMNH, Natural History Museum of Utah, Salt Lake City, Utah; USNM/NMNH, United States National Museum/National Museum of Natural History; YPM, Yale Peabody Museum, New Haven, Connecticut; ZMNH, Zhejiang Museum of Natural History, Hangzhou, China.

## 2 MATERIALS AND METHODS

### 2.1 Geological setting and Taphonomy

PEFO 31219 was recovered from the Petroglyph Phytosaur Site (PFV042) inside Petrified Forest National Park (Parker & Martz, 2010). It was found in the Petrified Forest Member of the Chinle Formation in a red siltstone bed marked by structures interpreted as slickensides, root traces, fine filled cracks, iron and manganese concentrations, small carbonate nodules, reduction spots, and apparent insect burrows. These structures are interpreted by Loughney et al. (2011) as evidence of a low sedimentation rate in a distal, well‐drained floodplain environment. Rasmussen et al. (2020) and Kent et al. (2018) determined a latest Norian age for the entire Petrified Forest Member using detrital zircon dating and correlation with the Newark‐Hartford astrochronostratigraphic polarity time scale, respectively. The stratigraphically lowest known occurrence of the phytosaur genus *Machaeroprosopus* occurs in the Jim Camp Wash beds of the underlying Sonsela Member, marking the lower boundary of the Revueltian holochronozone (Martz & Parker, 2017). Since the lowest known occurrence of a “*Redondasaurus*”‐type mystriosuchinine phytosaur (which marks the lowest occurrence of the Apachean holochronozone) occurs in the overlying Owl Rock Member at Ward's Terrace, Arizona (Martz & Parker, 2017), the entire Petrified Forest Member in Arizona can likely be assigned to the Revueltian holochronozone.

PEFO 31219 includes a complete skull, partial mandibles (Ballew, 1989), 22 presacral vertebrae, 3 sacral vertebrae, and 6 caudal vertebrae, pelvic elements, a complete left femur, osteoderms, and ribs. Assuming a pattern of hydrodynamic dispersal for bone elements similar to what has been demonstrated in modern crocodylians (Blob et al., 2022), it appears that little hydrodynamic dispersion of elements took place after death. The absence of most of the limb elements and the tail suggests the passage of some time for decay and the removal of these elements (de Araújo Júnior & da Silva Marinho, 2013; Syme & Salisbury, 2014; Toots, 1965), but not enough time for the skeleton to be completely disarticulated (Behrensmeyer, 1991).

### 2.2 Taxonomy

PEFO 31219, also known as the “Petroglyph Phytosaur” (William G. Parker, personal communication to Caleb N. LePore, 2023), was referred to as *Pseudopalatus buceros* by Ballew (1989). Though *P. buceros* and her newly described species *P. mccauleyi* have rostral crests, *P. buceros* was found to be more closely related to the uncrested form *P. pristinus* based on their (1) relatively small post‐temporal fenestrae, (2) squamosals that project relatively far posteriorly, terminating in short, knob‐like processes, (3) small anterior premaxillary teeth, and (4) a basioccipital that has a relatively small head and a relatively long neck. *P. mccauleyi* was distinguished by Ballew (1989) from *P. buceros* and *P. pristinus* in having (1) post‐temporal fenestrae enlarged by medial extension, (2) a triangular outline of the squamosal, (3) a basioccipital with a relatively large head and a relatively small neck, and (4) long, thin opisthotics. Long and Murry (1995) later separated pseudopalatine phytosaurs into two genera, reserving *Pseudopalatus* for *P. pristinus* and erecting *Arribasuchus* for *P. buceros* and *P. mccauleyi*. *Arribasuchus* was distinguished from *Pseudopalatus* by its heavy, partially crested skull, shorter rostrum, closely spaced, heterodont teeth, a dorsoventrally deeper posterior process of the squamosal that tapers to a blunt rather than pointed end, and a larger descending process of the squamosal. Long and Murry (1995) referred the “Petroglyph Phytosaur” to *A. buceros*. They were unconvinced that *Arribasuchus mccauleyi* should be separated from *A. buceros* based on the characters listed by Ballew (1989), but they nevertheless tentatively retained the species in their taxonomy. Hungerbühler (2002) later found *Arribasuchus buceros* and both species of *Pseudopalatus* to form a clade and considered the former genus to be a junior synonym of the latter. Parker and Irmis (2004) later assigned the Petroglyph Phytosaur to *P. mccauleyi* based on their comparison with the holotype skull of *P. mccauleyi* (UCMP 126999), finding that both skulls share the same squamosal and exoccipital morphology, position of the external nares relative to the skull roof, and degree of heterodonty. Stocker (2010, 2012) later used PEFO 31219 as a voucher specimen for *P. mccauleyi* alongside the holotype specimen (UCMP 126999) and recovered it in a clade with *P. pristinus* + *Mystriosuchus westphali*. *Pseudopalatus* was later found to be a junior synonym of *Machaeroprospus* by Parker et al. (2013). Jones and Butler (2018b) used PEFO 31219 as one of three specimens to score *Machaeroprosopus mccauleyi*. These authors note that the skull of PEFO 31219 possesses a squamosal with a posterior process that terminates in a short knob, unlike the holotype of *M. mccauleyi* (UCMP 126999), though the two skulls are greatly similar in all other respects. Unfortunately, no full‐length description of the skull of PEFO 31219 has yet been published, so we await a full reanalysis of the taxonomy of this specimen.

### 2.3 Descriptive methods

We described the three sacral vertebrae with their corresponding ribs, the last dorsal vertebra, and both ilia of PEFO 31219. We used the terminology of Wilson (1999) to describe vertebral laminae and the criteria of Nesbitt (2011) to identify the two primordial sacral vertebrae based on the morphology of their respective ribs and the position and morphology of their corresponding scars on the medial surfaces of the ilia. Once we established the identity of the two primordial vertebrae in the sacrum, we determined whether the third vertebra was incorporated into the sacrum from the dorsal series (added anteriorly), the caudal series (added posteriorly), or inserted between the two primordial vertebrae. We then compared the morphology of the sacral vertebrae, ribs, and ilia to those of other phytosaur specimens collected across the United States (see Table S1)—as well as specimens described by previous authors—to determine whether a caudosacral is present in any other phytosaur specimens. We also examined each specimen for evidence of a dorsosacral vertebra, so as to determine the frequency of this element's occurrence in phytosaurs. Following the description of Griffin et al. (2017), we consider ilium specimens to have a dorsosacral rib scar if they have a distinct, small scar on the medial ridge of the ilium anterior to the primordial first sacral rib scar.

All vertebral specimens were measured using VINCA DCLA‐0605 digital vernier calipers to the nearest 0.01 mm. The measurements are listed in the supplementary information. All vertebral, rib, and ilium specimens were photographed using a SONY ILCE‐6000, Canon EOS Rebel T6, or Canon PowerShot SX620 HS digital camera.

## 3 RESULTS

### 3.1 Sacral vertebrae and ribs

**FIGURE 1:**
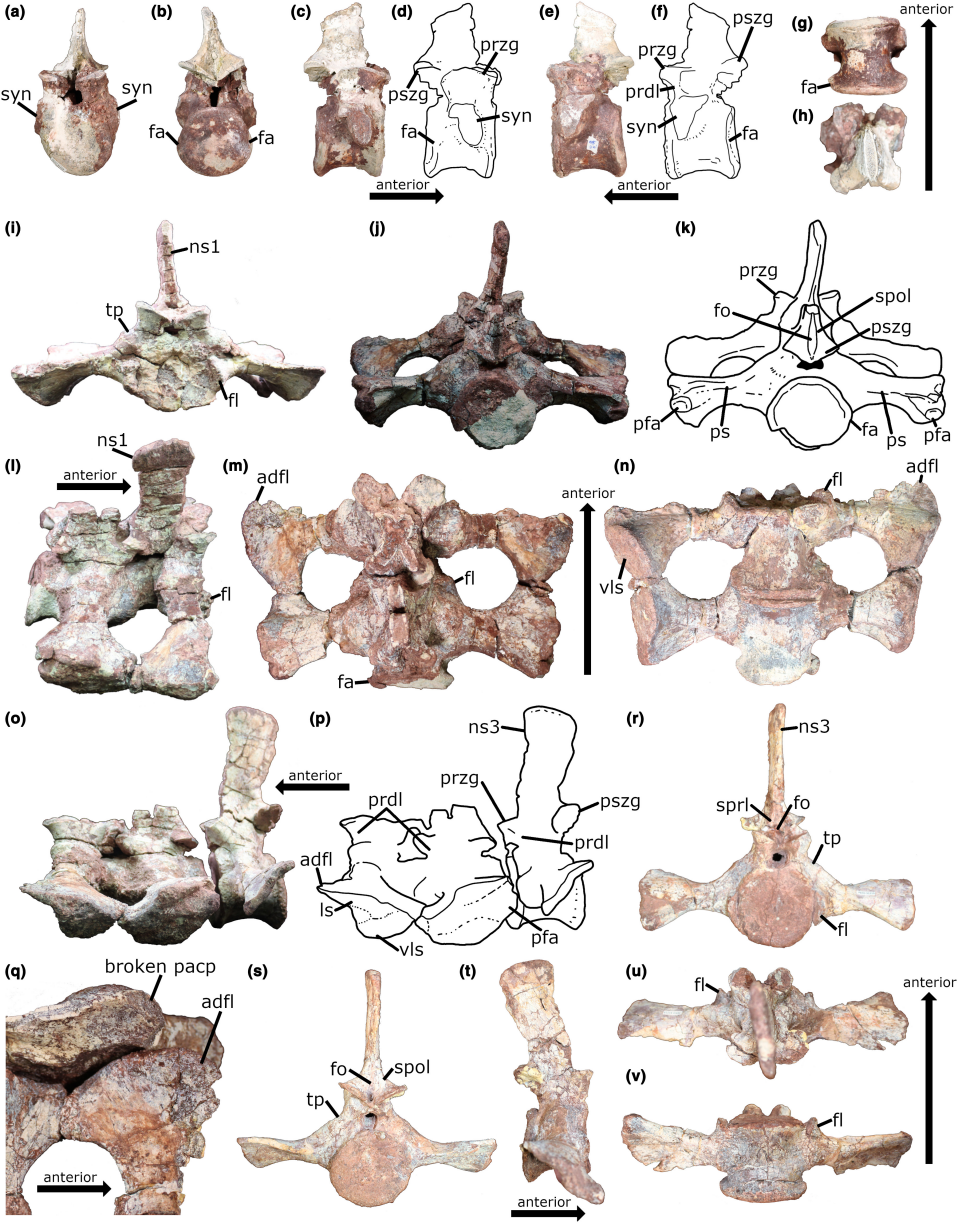
Sacral elements of PEFO 31219. (a) Posteriormost dorsal vertebra in anterior view and (b) posterior view. (c) Photograph and (d) line drawing of the last dorsal vertebra in right lateral view. (e) Photograph and (f) line drawing of the last dorsal vertebra in left lateral view. (g) Last dorsal vertebra in ventral view and (h) dorsal view. (i) First and second sacral vertebrae in anterior view. (j) Photograph and (k) line drawing of the first and second sacral vertebrae in posterior view. (l) First and second sacral vertebrae in right lateral, (m) dorsal, and (n) ventral views. (o) Photograph and (p) line drawing of the first and second sacral vertebrae with the caudosacral vertebra in left lateral view. (q) Up‐close dorsal view of the left first sacral rib in articulation with the ilium, highlighting the presence of a thin anterodorsal flange on the distal end of the rib. (r) Third sacral (caudosacral) vertebra in anterior, (s) posterior, (t) right lateral, (u) dorsal, and (v) ventral views. See Table S2 for measurements. adfl, anterodorsal flange of the first sacral rib; fa, facet; fl, flange; fo, fossa; ls, lateral surface for articulation with the ilium; ns1, neural spine of the first sacral vertebra; ns3, neural spine of the third sacral vertebra; pacp, preacetabular process; pfa, posterior facet for third sacral rib; prdl, prezygodiapophyseal lamina; przg, prezygapophysis; ps, posterior shelf; pszg, postzygapophysis; spol, spinopostzygapophyseal lamina; sprl, spinoprezygapophyseal lamina; syn, synapophysis; tp, transverse process; vls, ventrolateral surface for articulation with the ilium.

**FIGURE 2:**
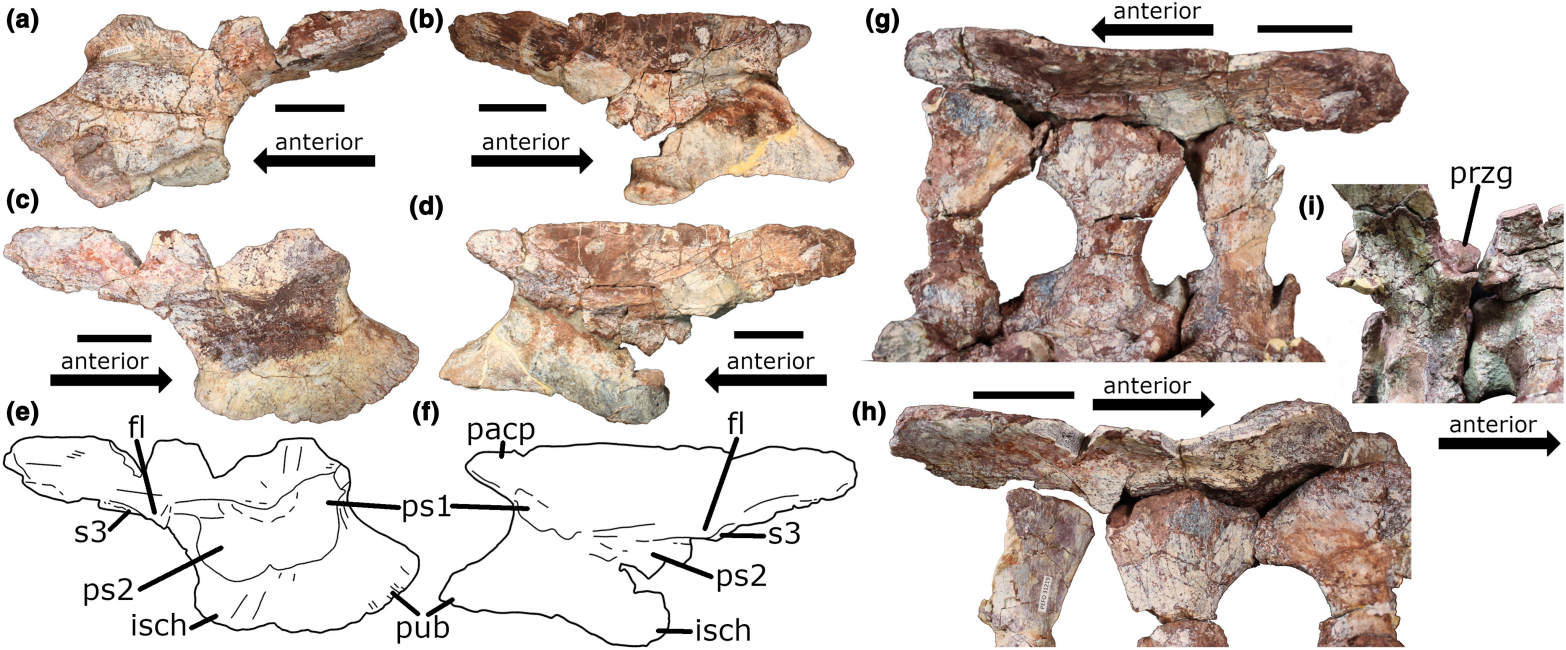
Ilia of PEFO 31219. (a, b) Left and right ilia, respectively, in lateral view. (c, d) Photographs and (e, f) line drawings of the left and right ilia, respectively, in medial view. (g) Right and (h) left ilia in articulation with the sacral ribs in dorsal view. (i) Third sacral (caudosacral) vertebra in articulation with the second sacral vertebra in right dorsolateral view. Scale bar: 5 cm. See Table S2 for vertebral measurements. fl, flange; isch, ischial peduncle; pacp, preacetabular process; przg, prezygapophysis; ps1, first primordial sacral rib scar; ps2, second primordial sacral rib scar; pub, pubic peduncle; s3, third sacral rib articulation surface.

PEFO 31219 possesses at least three sacral vertebrae (Figure 1). Each vertebra is sutured to a pair of large ribs that flare laterally and articulate with the medial surface of the ilium (Figure 2). The ribs of the first (S1) and second (S2) sacrals meet distally and appear to be fused to one another. The centra of S1 and S2 appear to be co‐ossified dorsally but not ventrally (Adam Marsh, personal communication to Caleb N. LePore, 2022). There is no fusion between either the ribs or the centra of S2 and the third sacral (S3).

The posteriormost dorsal vertebra is also preserved and may have been part of the sacrum (Figure 1a–e). The posterior face of the centrum has anteriorly beveled lateral edges that form facets (fa in Figure 1b,d,f,g) for the reception of the anteroproximal flanges of the ribs of S1 (fl in Figure 1i,l,n). These structures would have helped to stabilize the intervertebral joint. The lateral surfaces of the centrum and the short transverse processes of the neural arch bear a pair of robust rib facets that would have supported large ribs. Each rib facet consists of a combined parapophysis and diapophysis (i.e., synopophysis). Though the ribs are not preserved in articulation with the element, a remnant of the distal end of the left rib may be preserved as a dorsoventrally thin flange fused to the anterodistal edge of the left rib of S1 (adfl in Figure 1m,n,p,q). This flange possesses what appears to be an open suture (Adam Marsh, personal communication to Caleb N. LePore, 2022), which is consistent with it having originally been a separate element. Additionally, this flange would have articulated with the ilium along the medial side of the preacetabular process (Figure 1q), which is where a dorsosacral rib typically articulates in phytosaurs (Griffin et al., 2017). This may suggest that the sacrum of PEFO 31219 possessed a total of four vertebrae with their associated ribs. However, since we cannot determine this with certainty, we will refer to this specimen as possessing *at least* three sacral vertebrae.

The anterior surface of the centrum of S1 and the posterior surface of the centrum of S2 are shallowly concave. The apparent co‐ossification of the centra of S1 and S2 make it impossible to determine the shape of their contacting surfaces; thus, we cannot say with certainty that S1 and S2 are truly amphicoelous. Nevertheless, S3 is amphicoelous. The anterior face of the centrum of S1 is roughly circular in outline. It is wider than tall, even without the additional width supplied by the anteroproximal flanges of the first sacral ribs. Not counting these flanges, the width of the S1 centrum is approximately equal to its anteroposterior length (see Table S2 for measurements). The first sacral centrum is longer anteroposteriorly than any of the presacral vertebrae. The posterior articular face of S2 is circular, though its lateral margins are expanded laterally and beveled anteriorly for articulation with the anteroproximal flanges of the S3 ribs. The width of the posterior face of the centrum in S2 is greater than its height, approximately equaling the anteroposterior length of the centrum. The S2 centrum is also longer than S1. The posterior face of S2 is situated more ventrally than the anterior face of S1, indicating that these elements formed part of the downward curvature of the spine in the sacro‐caudal region. The anterior and posterior articular faces of the S3 centrum are circular (Figure 1n,o). The anterior face is taller than wide if the anteroproximal flanges of the ribs are excluded. The posterior articular face is slightly wider than tall. The posterior articular face appears to be positioned lower than the anterior face, indicating that this element also helped form the downward curvature of the spine. The height of the centrum is greater than its anteroposterior length, and its length is approximately equal to its width, excluding the ribs. The ventral surface of the centra of all three sacrals are flattened relative to the ventral surface of the last dorsal vertebra. No midline keels are present on any of the sacral centra. In all three sacrals, the anterior parts of the lateral surfaces of the centra are covered by the sacral ribs. The sutures between the ribs and the vertebrae are visible in all three sacrals (e.g., see Figure 1n,s). The neurocentral sutures are much harder to see, being partially covered by the ribs and mineral coatings.

The ribs of S1 project ventrolaterally at an angle of ~15° from horizontal in anterior view. In dorsal view, they curve and expand posteriorly towards their articulation with the S2 ribs. Their dorsal surfaces are relatively flat, while their ventral surfaces are convex anteroposteriorly. Their lateral ends are dorsoventrally thickened and possess two distinct surfaces for articulation with the ilium, as in *Nundasuchus songeaensis* (Nesbitt et al., 2014) and *Garjainia prima* (Maidment et al., 2020; see their figure 7). The laterally facing surface is dorsoventrally compressed, anteroposteriorly elongated, and tapering anteriorly and posteriorly (ls in Figure 1p). In lateral view, this surface, along with the entire rib, is angled along an anterodorsal‐posteroventral axis. Ventral to this lateral surface is a second surface that faces ventrolaterally (vls in Figure 1n,p). It is elliptical in outline, with its long axis directed posteroventromedially. In dorsal view, the distal margins of each rib are convex and deflected posteromedially (Figure 1m). Proximally, each rib has two heads that, while not separated, are nonetheless distinguishable. The anteroventral head is the capitulum, which is fused to the centrum, and the posterodorsal head is the tuberculum, which is fused to the laterally short, robust transverse process. The capitula form the anteroproximal flanges that project beyond the anterior surface of the centrum to articulate with the posterior face of the last dorsal centrum. In dorsal and ventral view, the large circular openings that separate the ribs of S1 and S2 anteroposteriorly are visible (Figure 1m,n).

The ribs of S2 are oriented ventrolaterally at an angle of ~10° to horizontal in posterior view (Figure 1j), which is less acute than that of the first sacral. At their distal ends, the ribs are expanded equally in the anterior and posterior directions (Figure 1m,n). In lateral view, the ribs dip anteroventrally posterodorsally (Figure 1l,o). At their distal ends, the dorsal surface of each rib is not flat, but rather, possesses a notable anteroposterior convexity at their anteroposterior middle. The ribs are dorsoventrally thick with ventral surfaces that are anteroposteriorly convex. The articular surface of each rib has an oval‐shaped, ventrolaterally facing anteroventral portion and a dorsoventrally thin, laterally directed posterodorsal portion (Figure 1o,p). Posterior to and continuous with the thickened anteroventral portion, there is a dorsoventrally thick section of bone that supports a posteriorly facing facet (pfa in Figure 1k,p) for articulation with the anterodistal tips of the S3 ribs. Proximally, the S2 ribs have the same double‐headed morphology as in the S1 ribs. Their capitular heads extend beyond the anterior face of the centrum to embrace the centrum of S1.

The ribs of S3 project horizontally in anterior and posterior view (Figure 1r,s). They are expanded anteroposteriorly at their distal ends (Figure 1u). Their posterodistal ends are broken, so it is impossible to know how far they extended posteriorly. The distal ends of the ribs are exceptionally thin dorsoventrally and are strongly angled along an anteroventral‐posterodorsal axis in lateral view (Figure 1o,p–t). The left rib is airfoil‐shaped in lateral view (Figure 1o,p), while the right rib is sigmoidal, with its anterior portion dorsally concave and its posterior portion dorsally convex. This asymmetry between the ribs is likely due to taphonomic distortion of the right rib. The anterodistal end of each rib forms a pointed tip that articulates with the corresponding facet on the posterodistal end of each S2 rib. The S3 ribs articulate with the medial shelf of the postacetabular process of the ilium (Figure 2g,h). Since this shelf extends along the full length of the postacetabular process, each S3 rib may have extended along the full length of the postacetabular process, as in the second sacral ribs of *Postosuchus kirkpatricki* (UMMP 7266; Weinbaum (2013), Case (1922)). The proximal ends of the ribs have the same double‐headed morphology seen in the sacral ribs of S1 and S2. The capitula of the ribs extend beyond the anterior face of the centrum as anteroproximal flanges (fl in Figure 1r,u,v).

In all three sacrals as well as the posteriormost dorsal, the transverse processes connect to the prezygapophyses by the prezygodiapophyseal laminae (prdl in Figure 1f,p). Though significantly damaged, the prezygapophyses of the posteriormost dorsal are estimated to have an angle of ~140°. The prezygapophyses of S1 have an angle of ~125° (Figure 1i) with articular surfaces that are elliptical and oriented anterolaterally in dorsal view (Figure 1m). The postzygapophyses of S2 have an angle of ~125° and are concave in posterior view (Figure 1j,k). The prezygapophyses of S3 have an angle of ~120° and are slightly convex in anterior view (Figure 1r). They are elliptical in dorsal view and are projected anterolaterally (Figure 1r), though they are missing their distal ends. The posterior part of the neural arch supporting the postzygapophyses of S2 has been taphonomically crushed dorsoventrally such that the prezygapophyses of S3 no longer fit beneath them (Figure 1o,p; 2I). The postzygapophyses of S3 have an angle of ~115°, project laterally, are concave in posterior view, and are wider mediolaterally than the prezygapophyses (Figure 1s). The postzygapophyses of both S1 and S2 are greatly reduced in size relative to those of the posteriormost dorsal, while the prezygapophyses of S2 and S3 are greatly reduced relative to those of the last dorsal and S1 (see Table S2 for measurements). Similarly, Camp (1930) described the zygapophyses of the first sacral in *Smilosuchus adamanensis* (UCMP 26699) as being smaller than those of the last dorsal, and the zygapophyses of the second sacral as being smaller than those of the first.

On the lower part of the neural spine in all three sacrals and the last dorsal of PEFO 31219, there is an oval‐shaped fossa medial to the spinopostzygapophyseal laminae (fo in Figure 1k,s). There also appears to be a shallow fossa on the lower part of the anterior surface of the spine between the spinoprezygapophyseal laminae in S3 (Figure 1r), which may also be present in S1 and S2 but cannot be definitively discerned from available photographs.

The entire heights of the neural spines are preserved in S1 and S3. (It should be noted that while the S1 neural spine is present in Figure 1i,l, it is not present in Figure 1m,o,p.) The spine of S2 is broken off at its base, though its size and morphology are inferred to have been intermediate to the spines of S1 and S3. The spine of S1 is more than 1.5 times the height of the centrum and is taller than all the presacral spines preserved. It ascends vertically with a slight posterior bend, expanding anteroposteriorly to more than two‐thirds the length of the centrum at its dorsal margin. The dorsal margin of the spine is convex and dips posteroventrally in lateral view. The spine of S3 is 1.9 times the height of the centrum and 1.3 times the height of the S1 spine. It expands anteroposteriorly at its dorsal end to a length of more than five‐sixths the length of the centrum. Overall, it projects slightly posteriorly and has a distinct sigmoid curvature in lateral view: the lower half curves slightly posteriorly and the upper half curves slightly anteriorly. The dorsal margin of the spine is convex and dips posteroventrally. In dorsal view, both spines are cigar‐shaped; neither are greatly expanded laterally at their dorsal apices.

### 3.2 Ilia

Both ilia are preserved in PEFO 31219. The right ilium is better preserved overall, though the medial surface and rib scars are better preserved in the left ilium (Figure 2c,f). The scar left by the first sacral rib is located on the dorsal part of the pubic peduncle where it joins with the preacetabular process. It is ovoid to rhomboid in shape, with the longer axis directed anterodorsally‐posteroventrally. The anterodorsal tip of this scar approaches the preacetabular process, and the anterior flange of the left S1 rib would necessarily have articulated with the preacetabular process medially. The preacetabular process, which is only preserved on the right ilium, is long enough to have articulated with such a flange. Unfortunately, this flange is not preserved in the right S1 rib and what is preserved of the right rib would not have continued anteriorly along the preacetabular process. The medial ridge of the ilium forms the dorsal margin of the S1 rib scar. Posteriorly, the S1 rib scar is continuous with the S2 scar. The S2 rib scar is comma‐shaped, being anteriorly rounded and posterodorsally thin and tapered. The majority of the rib scar is located on the dorsal part of the ischial peduncle, with only the “tail” of the “comma” barely extending onto the anteriormost part of the postacetabular process. As with the S1 rib scar, the medial ridge of the ilium forms the dorsal margin of the S2 rib scar. Posterior to the S2 rib scar, a thin flange of bone (fl in Figure 2e,f) projects medially from the medial ridge. The S3 rib articulates just posterior (and slightly ventral) to this flange.

## 4 DISCUSSION

### 4.1 Identification of the three sacral elements

**FIGURE 3:**
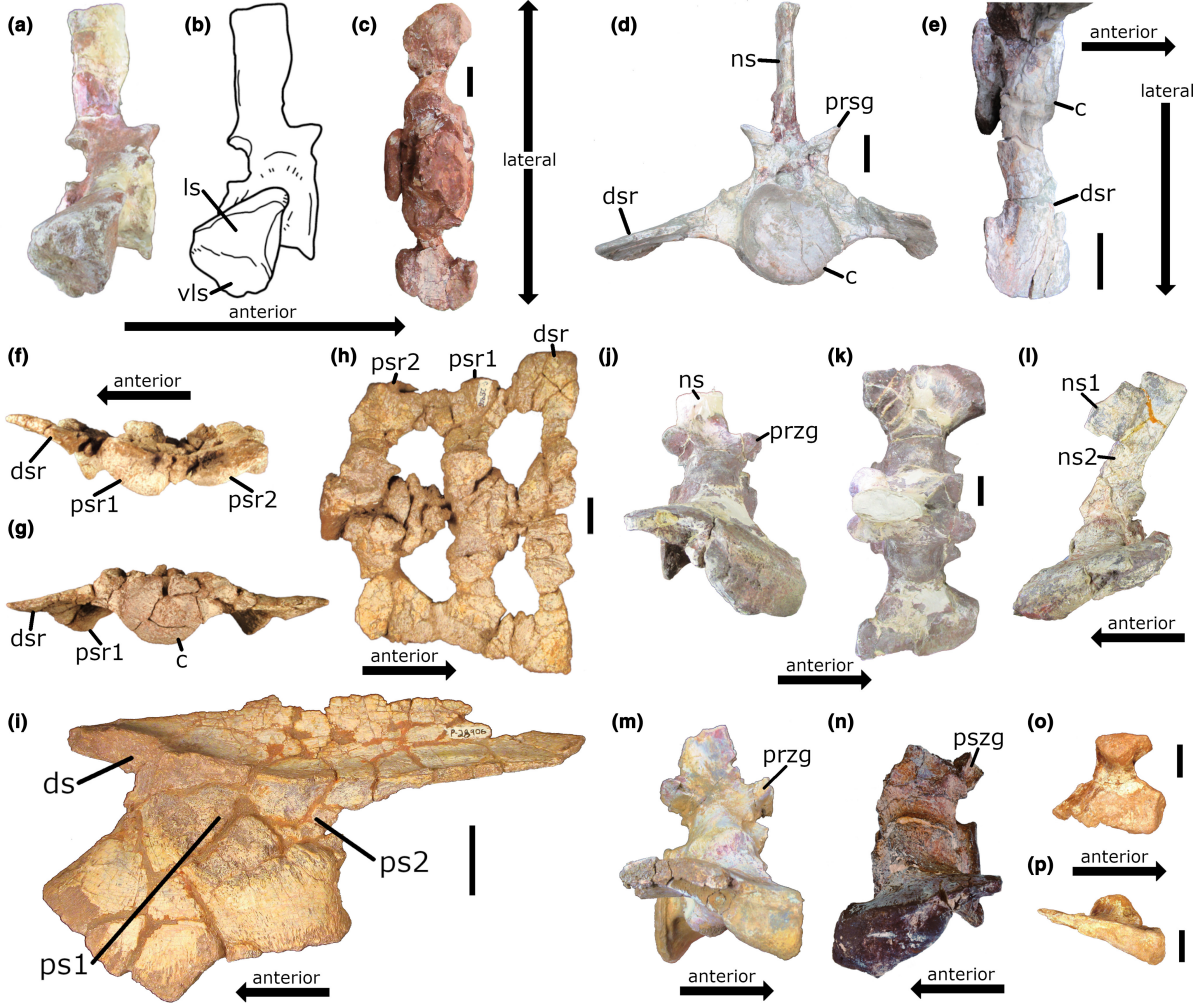
Phytosaur sacral elements. (a) Photograph and (b) line drawing of the first sacral vertebra and ribs from UCMP 27036 (holotype of “*Machaeroprosopus*” *zunii*) in right lateral view (interzygapophyseal length = 94.1 mm). (c) First sacral vertebra with ribs from MCCDM 1748 in dorsal view. (d) Dorsosacral vertebra with ribs from TTU P14944 in anterior view. (e) Right dorsosacral rib from TTU P14944 in right dorsolateral view. (f) Dorsosacral and primordial first and second sacral vertebrae with ribs from NMMNH P‐28906 in left lateral, (g) anterior, and (h) dorsal views (centrum width of dorsosacral = 59.3 mm; length of the left first primordial sacral rib at distal end = 65.9 mm). (i) Right ilium of NMMNH P‐28906 in medial view. (j) Second sacral vertebra and ribs from UCMP 27036 in right lateral and (k) dorsal view (interzygapophyseal length = 92.4 mm). (l) Second sacral vertebra with ribs from UCMP 26699 (holotype of *Smilosuchus adamanensis*) in left lateral view (neural spine length [ns2] = 43.6 mm). (m) Isolated second sacral vertebra and ribs (TTU P11351) in right lateral view (interzygapophyseal length = 86.3 mm). (n) Isolated second sacral vertebra with ribs (UMMP 13762) in right lateral view (centrum length = 61.5 mm; length of left rib at distal end = 89.1 mm). (o) Second sacral rib from NMMNH P‐20852 (*Machaerprosopus buceros*) in dorsal and (p) right lateral views. Scale bar: 3 cm. c, centrum; ds, dorsosacral rib scar; dsr, dorsosacral rib; ls, lateral surface for articulation with the ilium; ns, neural spine; ns1, neural spine of the first sacral vertebra; ns2, neural spine of the second sacral vertebra; przg, prezygapophysis; ps1, first primordial sacral rib scar; ps2, second primordial sacral rib scar; psr1, first primordial sacral rib; psr2, second primordial sacral rib; pszg, postzygapophysis; vls, ventrolateral surface for articulation with the ilium.

Nesbitt (2011) described the primordial first sacral rib of non‐archosaurian archosauriforms and most crocodylian‐line archosaurs as massive and circular in lateral view. It usually articulates with the ilium on the dorsal part of the pubic peduncle where it joins with the preacetabular process. This is consistent with published descriptions and depictions of phytosaur sacra in the literature. *Parasuchus hislopi*, from the illustrations of Chatterjee (1978, figure 8o,p), appears to have this type of first sacral rib morphology. *Angistorhinus* has similar, massive first sacral ribs, as depicted in figure 4b of Lucas et al. (2002). McGregor (1906, Plate X, figure 40) depicts a vertebra with this same morphology in *Rutiodon carolinensis*, though he mislabeled it as a second sacral (as previously noted by von Huene [1922] and Griffin et al. [2017]). The description of Nesbitt (2011) is consistent with all phytosaur specimens we examined wherein the first sacral could be clearly identified based on its position within the vertebral column (UCMP 26699, UCMP 27036) or through articulation with the medial surfaces of the ilia (NMMNH P‐29806, PEFO 34852). Isolated elements were easily identified as first sacral ribs/vertebrae using the criteria of Nesbitt (2011). In all ilia examined, the location of the scar left by the first primordial sacral is identical to that of the description by Nesbitt (2011) summarized above. Based on these same characteristics observed in S1 and the medial ilium of PEFO 31219, we identify S1 of PEFO 31219 as the primordial first sacral vertebra. Additional characters seen in other specimens that support this identification include: (1) the dorsal surface of the rib is oriented along an anterodorsal‐posteroventral axis (e.g., UCMP 27036; see Figure 3a,b); (2) the first sacral ribs have two distinct articular surfaces, namely a dorsal surface that faces laterally and a ventral surface that faces ventrolaterally (Figure 3a,b); (3) the distal margins of the ribs are deflected posteromedially in dorsal view (e.g., MCCDM 1748 in Figure 3c).

Nesbitt (2011) noted that in some taxa (e.g., several poposauroids), an anterodorsal expansion of the primordial first sacral rib articulates with the preacetabular process of the ilium. The flange present on the left rib of S1 in PEFO 31219 may correspond to such an expansion. Alternatively, it could be the remnant of a dorsosacral rib. Dorsosacral ribs in phytosaurs are dorsoventrally flattened at their distal ends (Figure 3d–g), articulate posterodistally with the primordial first sacral ribs (Figure 3h), and articulate with the ilium on the medial side of the preacetabular process (Figure 3i). The flange of the first primordial sacral rib of PEFO 31219 is consistent with this morphology. In most of the ilia we observed that did not have associated dorsosacral ribs, there is small surface anterodorsal to the first sacral rib scar that we identify as a dorsosacral rib scar (see discussion below).

In identifying the second primordial sacral vertebra, Nesbitt (2011) described the second sacral rib as being more massive than the first sacral rib, posteriorly expanded distally, and having a teardrop‐shaped articular surface for the ilium. The matching rib scar on the medial ilium has a more massive anterior part that articulates with the medial side of the ischial peduncle, while the thinner posterior part sits below an anteroposteriorly running ridge on the medial side of the postacetabular process. *Parasuchus hislopi* is depicted as having such a second sacral rib by Chatterjee (1978) and a rib of this morphology can be inferred to have been present in *Rutiodon carolinensis* from the shape of the primordial second sacral rib scar on the medial face of the ilium (see Plate X, figure 41a, of McGregor (1906)). The specimens we observed also match this description, allowing for easy identification of isolated elements. They are airfoil‐shaped in lateral view, being anteriorly thick, posteriorly thin, dipping anteroventrally‐posterodorsally, and arched anteroposteriorly. There is some variation in the distal thickness of the ribs, being thicker in UCMP 27036 (Figure 3j,k), UCMP 26699 (Figure 3l), TTU P11351 (Figure 3m), and UMMP 13762 (Figure 3n) and thinner in PEFO 34852 (see figure 2 in Griffin et al. [2017]) and NMMNH P‐20852 (Figure 3o,p). In all cases where not still connected with the primordial first sacral ribs, the anterodistal corner of the ribs have a large, distinct facet for articulating with the primordial first sacral ribs. In stark contrast to PEFO 31219, none of the other specimens we observed or that have been described in the literature have a facet for a third sacral rib on the posterodistal ends of the S2 ribs. Further, the general morphology of the S2 ribs in PEFO 31219 in lateral view is not teardrop‐shaped, but rather, massive and rounded like in the S1 ribs. Nevertheless, other aspects of the morphology of this rib favor its identification as a primordial second sacral rib. Its dorsal surface is oriented on an anteroventral‐posterodorsal axis in lateral view (see Figure 1l), as in all other phytosaur second sacral rib specimens observed (compare with Figure 3j,l,n,p). Additionally, when the facet for the S3 ribs is disregarded, the S2 ribs of PEFO 31219 do appear to taper posterodorsally, with the posterior part of this rib consisting only of a dorsoventrally thin shelf. This is reflected in the “comma‐shaped” S2 rib scar on the medial surface of the ilium (Figure 2c,e). However, the ribs of S3 in PEFO 31219 do have a similar morphology to the second sacral ribs of PEFO 34852, being very dorsoventrally thin distally and strongly anteroposteriorly arched in lateral view. Additionally, the distal ends of the ribs are oriented along an anteroventral‐posterodorsal axis in lateral view. Could the third sacral of PEFO 31219 be the primordial second sacral and the second sacral be an insertion between the two primordial sacral vertebrae? Nesbitt (2011) identified the second sacral vertebra of the poposauroid *Arizonasaurus babbitti* as an insertion between the first and third (second primordial) sacral vertebrae based on the unique morphology of its sacral ribs. Unlike typical second sacral ribs in archosauriforms, they are not posteriorly tapered in *A. babbitti*, nor do they articulate with the ilium at the junction of the ischial peduncle and the postacetabular process. Nesbitt (2011) considered the loricatan *Batrachotomus kupferzellensis* to have had the same condition. In the case of *A. babbitti*, he made the observation that the area of articulation between the sacral ribs and the ilium is no greater than the area of articulation between these elements in phytosaurs, which possess only two sacral vertebrae. Additionally, the anterior extent of the first sacral ribs and the posterior extent of the third sacral ribs are reduced in *A. babbitti* relative to the two sacral ribs in the archosauriform *Euparkeria capensis*, apparently to make room for the inserted second rib in the former. Nesbitt (2011) interpreted these features as evidence that the additional sacral vertebra was inserted between the two primordial sacral vertebrae. Nevertheless, the second sacral vertebra in PEFO 31219 is unlikely to be an insertion given the morphology of the S2 rib, along with the placement of its corresponding scar on the medial surface of the ilium. Though the rib itself has a robust appearance in lateral view that superficially does not appear to be tapered posteriorly, the second sacral rib scar is comma‐ or teardrop‐shaped and is located at the junction of the postacetabular process and the ischial peduncle. This shape and position is typical for a primordial second sacral rib scar, strongly suggesting that S2 is, in fact, the primordial second sacral vertebra, not an insertion. The location of the rib scar for S3 is exclusively on the postacetabular process, not at all touching the ischial peduncle, which is consistent with it being an addition from the caudal series. Thus, we regard the S2 and S3 sacral vertebrae and ribs as the second primordial sacral and sacralized first caudal (caudosacral), respectively.

### 4.2 Development of the caudosacral in PEFO 31219

This is the first definitive description of a caudosacral vertebra in a phytosaur (though see below for a discussion of a possible occurrence in USNM 18313, *Smilosuchus gregorii*). Though some taphonomic distortion has occurred in this specimen as noted above, this distortion does not explain the observable connection between the ribs of the caudosacral and the medial ilia. The presence of a facet for the caudosacral ribs on the ventral side of the posterodistal ends of the S2 ribs clearly indicate that these two pairs of ribs met each other in life. Furthermore, the shape of the distal end of each caudosacral rib exactly matches the outline of the anteroventral margin of the postacetabular process, which is where they articulate with the ilium. The addition of the first caudal to the sacrum is not likely the result of pathology either, as there is no evidence of pathology on the external surface of the bone (e.g., no abnormal sutures, no external modifications to the bone texture, no reabsorption, and no missing features). Their perfect symmetry and precise articulation with the second primordial sacral ribs suggest they are a product of normal development.

The number and regional identity of elements in the vertebral column are controlled developmentally by two processes (Müller et al., 2010). The first process is somitogenesis. During development, somites (the developmental precursors of vertebrae) are formed periodically from the anterior end of the presomitic mesoderm in the embryo. According to the “Clock and Wavefront” model of somitogenesis, cells within the presomitic mesoderm undergo oscillations in gene expression, resulting in periodic “waves” of expression traveling from the posterior end to the anterior end of the presomitic mesoderm (Dequéant & Pourquié, 2008; Venzin & Oates, 2020; Yabe & Takada, 2016). Where these waves halt marks where the next somite will bud off from the presomitic mesoderm. Because of its periodicity, this oscillatory process is called the “segmentation clock.” The faster the “ticking” of this segmentation clock, the greater the number of resulting somites (and thus, vertebrae; Gomez et al., 2008). The second process involves the regional determination of the somites, which is accomplished through the activation of *Hox* genes. Different *Hox* genes are expressed in different segments of the developing embryo, determining the regional boundaries of the vertebral series (Burke et al., 1995; Gaunt, 1994). Alterations in the timing of *Hoxd‐11* gene expression have been shown to cause anterior or posterior shifts in the sacral series (Zákány et al., 1997). Griffin et al. (2017) argued that because changes in *Hoxd‐11* expression can affect the sacral region while not affecting other areas under the influence of these genes, and because sacral numbers can vary intraspecifically in extant organisms with no apparent deleterious effect, then variation in sacral number may represent a highly evolvable system. Small changes during development could result in considerable, non‐deleterious variability within a species. Griffin et al. (2017) proposed that changes in *Hox* gene regulation may explain the independent incorporation of a dorsosacral vertebra into the sacrum of multiple Triassic archosauriform lineages (including phytosaurs). This mechanism could also explain the acquisition of a caudosacral vertebra in PEFO 31219. Alternatively, a higher rate of “ticking” of the segmentation clock could cause more somites to be present in the region that would become the sacrum, thereby increasing the vertebral count in that region.

### 4.3 Variation in sacral vertebra count in phytosaurs

Unfortunately, no other sacra are known from *Machaeroprosopus mccauleyi*, so it cannot be determined whether all members of this species possessed caudosacral vertebrae. Thus, we cannot determine whether this trait was taxon‐specific or subject to intraspecific or sexual variation. A caudosacral vertebra has only been reported in one other phytosaur specimen, USNM 18313, the brachyrostral leptosuchomorph *Smilosuchus gregorii*. This specimen was noted by Heckert et al. (2021) as having two sacral vertebrae and one “transitional sacro‐caudal” vertebra. According to personal communication with one of the authors (Matthew Carrano, 2022), this element was labeled a “sacro‐caudal” vertebra based on the expanded and robust morphology of its transverse process. We were unable to verify whether this element articulates with the ilium, so we regard this specimen as possibly – but not definitively – having a caudosacral vertebra. However, from viewing a photograph of the medial side of the left ilium of this specimen on the NMNH Paleobiology Collections website (http://collections.si.edu/search/detail/edanmdm:nmnhpaleobiology_3451097), it does appear that USNM 18313 possesses a dorsosacral rib scar.

In PEFO 31219, the point of articulation for the distal end of the caudosacral rib on the medial surface of the ilium is not marked by a well‐defined scar as with the primordial first and second sacral vertebrae. Thus, the presence of a caudosacral rib articulation is likely to be undetected when examining only the medial surface of the ilium in phytosaurs. Likewise, if the distal ends of the ribs are missing from a first caudal, it may not be possible to determine whether it played a role in the sacrum. Careful excavation, preparation, and descriptions of well‐preserved, taxonomically identifiable phytosaur specimens are needed in order to determine the prevalence of this character in phytosaurs.

Prior to Griffin et al. (2017), most descriptions of postcranial material from phytosaurs (Camp, 1930; Chatterjee, 1978; Gozzi & Rensto, 2003; Lucas et al., 2002; McGregor, 1906) considered the number of sacral vertebrae in phytosaurs to be limited to two–the plesiomorphic condition in Archosauriformes (Nesbitt, 2011). One exception is McQuilkin (1998), who described a phytosaur skeleton from the Dockum Group of Texas as having three sacral vertebrae. Griffin et al. (2017) later demonstrated the presence of a dorsosacral vertebra in PEFO 34852 (referred to *Smilosuchus adamanensis*, but see Jones and Butler (2018b)), “*Redondasaurus”* cf. *gregorii* (MCCDM 1743‐1 and MCCDM 1743‐2), and a specimen of *Machaeroprosopus* from eastern Arizona (USNM 15860). They also argued for its presence in the holotype of *Smilosuchus adamanensis* (UCMP 26699) based on the presence of a flattened area on the anterodistal ends of the primordial first sacral ribs for articulation with the posterodistal ends of dorsosacral ribs and a scar on the medial ridge of the ilium anterior to the first primordial rib scar. Additionally, they suggested that the holotype of “*Machaeroprosopus*” *zunii* (UCMP 27036) may have had a dorsosacral based on its possession of anteriorly facing flanges formed by the anteroproximal portions of the primordial first sacral ribs, which articulate with corresponding facets on the posterior end of the centrum of the posteriormost dorsal. However, the posteriormost dorsal of “*M*.” *zunii* lacks associated ribs (personal observation). We were unable to examine the preserved ilium material of UCMP 27036 described by Camp (1930) for the presence of a dorsosacral rib scar.

**FIGURE 4:**
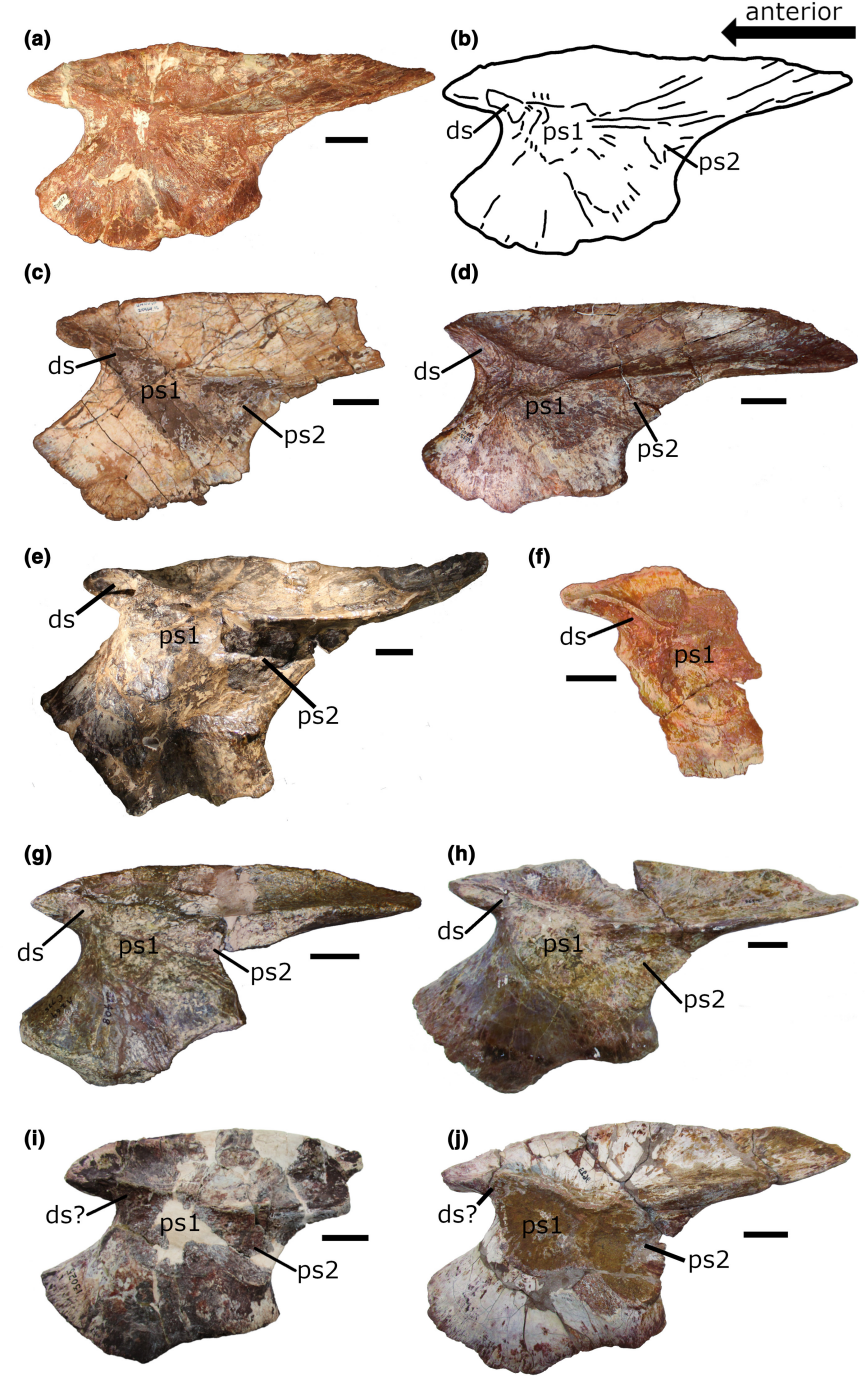
Phytosaur ilia in medial view. (a) Photograph and (b) line drawing of the right ilium of *Machaeroprosopus buceros* (NMMNH P‐20852). (c) Right ilium of “*Redondasaurus”* (UMNH VP 26654.16). (d) Right ilium of “*Redondasaurus” gregorii* (UCMP 65267). (e) Right ilium of *Smilosuchus gregorii* (AMNH FARB 3060). (f) Right ilium fragment from an indeterminate phytosaur (NMMNH P‐17874). (g) Right ilium of an indeterminate phytosaur (UCMP 32408). (h) Right ilium of an indeterminate phytosaur (UMMP 9624). (i) Right ilium of an indeterminate phytosaur (UMMP 13025). (j) Left ilium (mirrored) of an indeterminate phytosaur (UMMP 9623). Scale bar: 3 cm. ds, dorsosacral rib scar; ps1, first primordial sacral rib scar; ps2, second primordial sacral rib scar.

**FIGURE 5:**
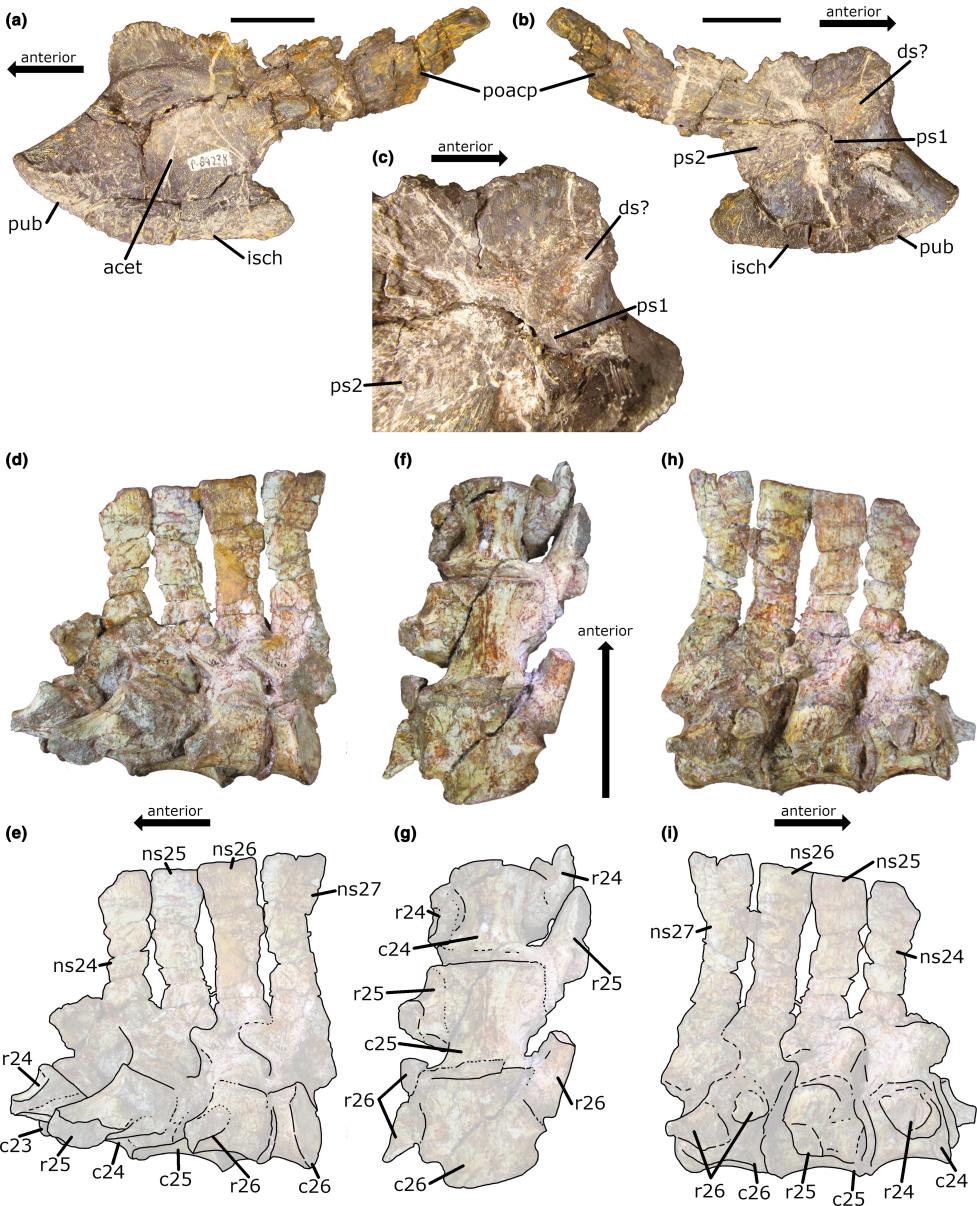
Ilium and sacral region of *Machaeroprosopus buceros/pristinus*. (a) Left ilium of *M. buceros* from the Snyder Quarry (NMMNH P‐69238) in lateral, (b) medial, and (c) close‐up medial views. (d) Twenty‐fourth through twenty‐seventh vertebrae of *M. pristinus* from the Canjilon Quarry (UCMP 34253) in left lateral, (e) ventral, and (f) right lateral views. Scale bar: 5 cm. Centrum lengths: see Table S3. acet, acetabulum; c23, fragment of centrum of vertebra 23; c24, centrum of vertebra 24; c25, centrum of vertebra 25; c26, centrum of vertebra 26; ds, dorsosacral rib scar; isch, ischial peduncle; ns24, neural spine of vertebra 24; ns25, neural spine of vertebra 25; ns26, neural spine of vertebra 26; ns27, neural spine of vertebra 27; pub, pubic peduncle; r24, rib of vertebra 24; r25, rib of vertebra 25; r26, rib of vertebra 26.

From our first‐hand examination of phytosaur sacra and ilia material, the occurrence of a dorsosacral vertebra appears to be common in phytosaurs. To the list of specimens with definitive dorsosacral ribs, we add NMMNH P‐28906 (Figure 3f–i) from the Chinle Formation of New Mexico, which was referred to *Smilosuchus* by Heckert (1999) based on characters of the ilium, and TTU P14494 (Figure 3d,e), an indeterminate phytosaur specimen from the upper unit of the Cooper Canyon Formation (Dockum Group) of Texas. Among the ilia specimens we examined, nearly all had surfaces on the medial sides of their preacetabular processes that we interpret as dorsosacral rib scars. These include a specimen referred to *Machaeroprosopus buceros* (Hunt, 1991) from the lower Bull Canyon Formation of New Mexico (NMMNH P‐20852; Figure 4a,b), a specimen of “*Redondasaurus*” from the Chinle Formation of Utah (UMNH.VP.25564; Figure 4c), a specimen referred to “*Redondasaurus*” *gregorii* by Spielmann and Lucas (2012) from the upper Redonda Formation of New Mexico (UCMP 65267; Figure 4d), an enormous specimen of *Smilosuchus gregorii* (AMNH FARB 3060; Figure 4e) from the Chinle Formation of Arizona (Colbert, 1947), an indeterminate phytosaur specimen from the Garita Creek Formation of New Mexico (NMMNH P‐17874; Figure 4f), two right ilia from the *Placerias* Quarry in the Chinle Formation of Arizona (UCMP 25956 and UCMP 32408; the latter is figured in Figure 4g), and an isolated ilium from the Dockum Group of Texas (UMMP 9624; Figure 4h). Additionally, several specimens we examined have surfaces on the medial sides of the preacetabular process that appear to be dorsosacral rib scars, but of which we are less confident. These include UCMP 25973 from the *Placerias* Quarry (not figured), along with two isolated ilia from the Dockum Group (UMMP 13025 and UMMP 9623; Figure 4i,j), and a specimen of *Machaeroprosopus buceros* from the Snyder Quarry of New Mexico (NMMNH P‐69238; Figure 5a–c). Lucas et al. (2016) described a partial ilium (PPHM WT 2927) from the Rotten Hill bonebed in the Tecovas Formation of the Dockum Group, which also appears to possess a dorsosacral rib scar (see figure 19C in Lucas et al., 2016). The Rotten Hill bonebed is close to Sierrita de la Cruz (“Sweetly Cruize”) Creek in Potter County, TX, which is where UMMP 13025 was collected. The two other UMMP ilia we observed (UMMP 9623 and UMMP 9624) closely resemble a large ilium (UMMP 7244, which we were unable to examine in person) depicted by Case (1922; Plate 13, figure B) that also appears to possess a dorsosacral rib scar. Case (1922) incorrectly identified the dorsosacral rib scar as the primordial first sacral rib scar. This specimen, along with UMMP 9623 and UMMP 9624, were collected from Crosby County, TX.

It is significant that we found evidence for a dorsosacral rib scar in *Machaeroprosopus buceros* (NMMNH P‐20852, NMMNH P‐69238), because the closely related *Machaeroprosopus pristinus* was described by Nesbitt (2011) as having only two sacral vertebrae. Nesbitt (2011) based this judgment on UCMP 34253, an articulated vertebral column collected from the Canjilon Quarry in the Chinle Formation of New Mexico. No skull was found with this specimen, and since the Canjilon Quarry preserves material from both *Machaeroprosopus buceros* and *Machaeroprosopus pristinus* (Ballew, 1989; Nesbitt & Stocker, 2008), this specimen may be attributed to either species. Furthermore, some workers consider *M. buceros* and *M. pristinus* to be synonymous, representing male and female morphs, respectively, of the same species (Zeigler et al., 2002). In examining UCMP 34253, we found that the 24th through 26th vertebrae of this specimen possess large ribs that are preserved in articulation with the centrum and the laterally short, anteroposteriorly broad transverse processes of each vertebra (Figure 5d–i). All of the ribs are broken distally and are taphonomically distorted, making attempts to identify them especially difficult. Though broken, the distal end of the left rib belonging to V25 is dorsoventrally thickened (r25 in Figure 5e), which indicates that it may belong to one of the primordial sacral vertebrae rather than a dorsosacral, as dorsosacral ribs in phytosaurs are dorsoventrally compressed distally (Griffin et al., 2017; this study). Anteroproximal flanges are visible on the right rib, which, when complete, may have reached the posterior centrum of the preceding vertebra (V24). In ventral and right lateral views (Figure 5f–i), the lateral edges of the posterior face of the centrum in V24 appear to be pushed forward to accommodate these flanges. In ventral view, V24 and V25 appear to have mediolaterally rounded centra, while V26 appears relatively flat. In phytosaurs, the primordial second sacral centrum is generally flatter ventrally than the primordial first sacral and dorsal centra. This feature supports the identification of V26 as the primordial second sacral vertebra. However, because no ilium was preserved, it is not possible to determine definitively which vertebrae articulated via ribs with the ilia. Thus, it cannot be ruled out that a dorsosacral vertebra may have been present in this specimen.

**FIGURE 6:**
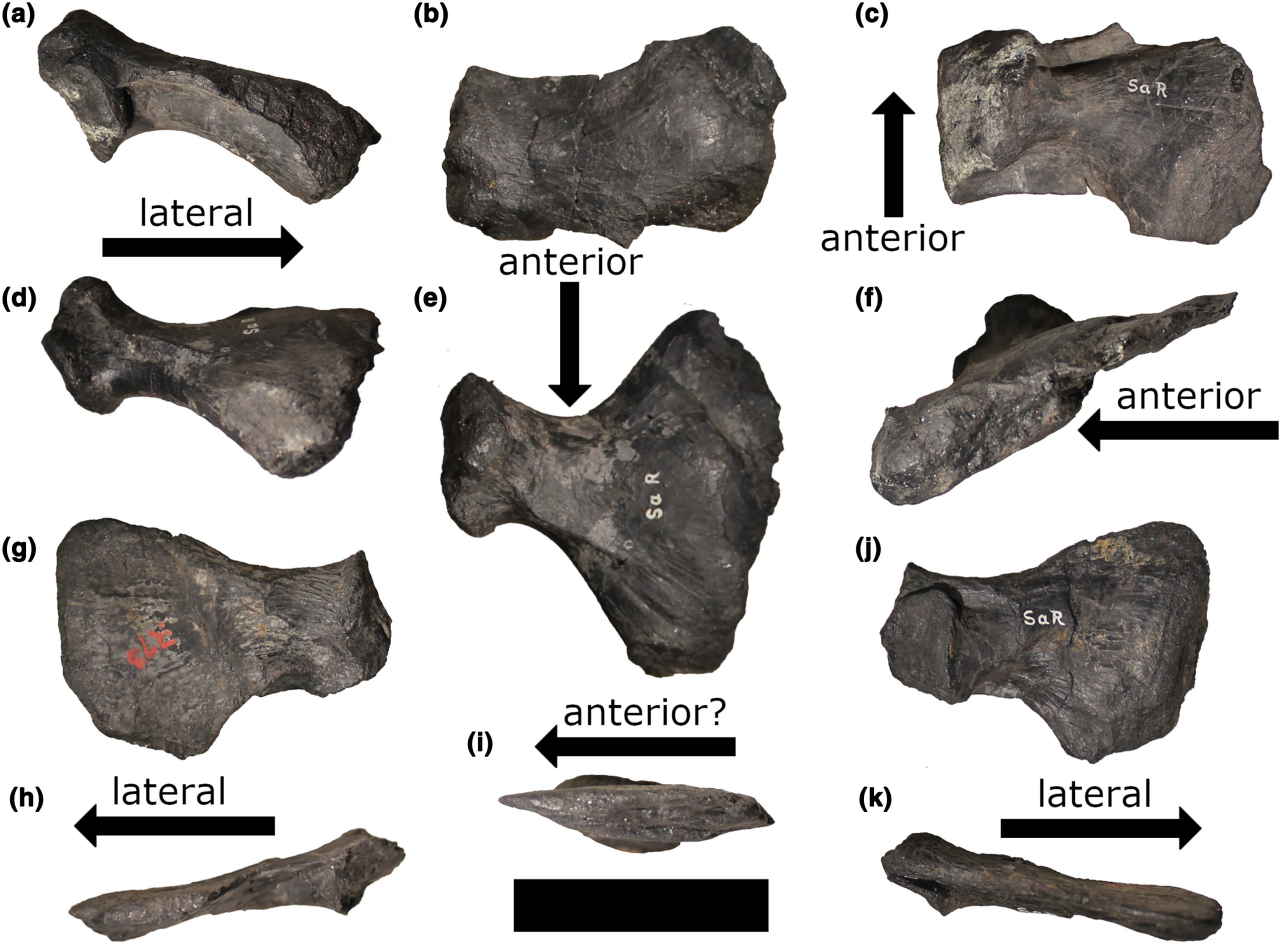
Sacral ribs of *Rutiodon carolinensis* (AMNH FARB 1). First sacral rib morphotype in (a) anterior, (b) dorsal, and (c) and ventral views. Second sacral rib morphotype in (d) anterior, (e) dorsal, and (f) lateral views. Third sacral (?) rib morphotype in (g) dorsal, (h) anterior (?), (i) lateral, (j) ventral, and posterior (?) views (k). Scale bar = 5 cm.

Like *Machaeroprosopus pristinus*, *Rutiodon carolinensis* (AMNH FARB 1) has been described as having two sacral vertebrae (McGregor, 1906). From examining McGregor's (1906) depiction of the right ilium of *R. carolinensis* in medial view (Plate X, figure 41a), there appears to be a flattened surface on the medial side of the preacetabular process that could be a dorsosacral rib scar. McGregor (1906) also notes the presence of facets on the proximal ends of the sacral ribs (labeled “X” in Plate X, figure 40a) which articulated with the beveled lateral edges of the posterior end of the centrum in the last presacral vertebra (“X” in Plate VIII, figure 16). According to McGregor (1906), in the posteriormost presacral vertebra, the proximal end of the left rib is ankylosed with the vertebra. Unfortunately, the distal end of the rib is missing, making it impossible to know whether it functioned as a dorsosacral rib. Although we were able to examine AMNH FARB 1, we did not take off the glass case it currently resides in, and thus we were unable to examine the medial surface of the ilium. We were able to examine a series of sacral (?) ribs from this specimen in the AMNH collection and identify two of them as the left primordial first (Figure 6a–c) and second (Figure 6d–f) sacral ribs. The third rib is different in morphology from the other two (Figure 6g–k), though it may actually be a caudal rib. A large partial skeleton of a phytosaur ascribed to *Rutiodon* (YPM VPPU 011544) from the red shales of the Triassic New Oxford Formation of Pennsylvania (Kochanov & Sullivan, 1994) includes both ilia, the primordial first sacral and the last presacral vertebrae, both with fused ribs (Sinclair, 1918). This specimen was referred to *Rutiodon* “*manhattanensis*” by Sinclair (1918) based on similarities in the ilia and femur with the partial skeleton AMNH FARB 4991 described by von Huene (1913). The last presacral vertebra, as figured and described by Sinclair (1918; see his figure 2), has a clear facet on the posterior face of the centrum for the reception of the anteroproximal flanges on the primordial first sacral ribs. Additionally, the last presacral rib is flattened distally and curves posteriorly, articulating with the anterodistal end of the primordial first sacral rib. Although Sinclair (1918) mentioned only two articular surfaces for sacral ribs on the medial surface of the ilium, these features strongly suggest that this specimen possesses a dorsosacral rib. We are currently working to determine definitively whether this specimen (and other possible *Rutiodon* specimens) possessed more than two sacral vertebrae.

*Colossosuchus techniensis* (Datta & Ray, 2023), placed within Mystriosuchinae outside of Leptosuchomorpha, was described as having a dorsosacral vertebra based on the presence of a dorsosacral rib scar on the medial ilium.

The short‐snouted Middle Triassic form *Diandongosuchus fuyuanensis* from the Falang Formation of eastern Yunnan Province, China (ZMNH M8770) was originally classified as a basal poposauroid by Li et al. (2012) but was reassigned to a basal position within Phytosauria by Stocker et al. (2017). The latter authors describe it as possessing three sacral vertebrae. Griffin et al. (2017) regarded this specimen as “dorsosacral ambiguous.” Based on figure 6a in Li et al. (2012) and figure 2 of Stocker et al. (2017), it appears that the third sacral rib is broad proximally, constricted mid‐length, and more anteroposteriorly expanded distally. The overall morphology of this rib strongly resembles that of a phytosaur primordial second sacral rib. The ribs belonging to the second vertebra of the sacrum of *D. fuyuanensis* are largely covered by other elements, though the proximal end of the right rib is visible in ventral view. The rib belonging to the first vertebra is narrow anteroposteriorly at its proximal end. It expands distally, while curving posteriorly, as in phytosaur dorsosacral ribs. The overall morphology of the two well‐exposed ribs (first and third) is very close to that of the “*Redondasaurus*” (MCCDM 1743‐1) sacral ribs figured by Griffin et al. (2017; figure 7). This is very curious, given that *D. fuyuanensis* occupies the basalmost position in Phytosauria and “*Redondasaurus*” occupies a highly derived position in all recent phylogenetic analyses (e.g., Butler et al., 2019; Jones & Butler, 2018a). This could suggest that the possession of a dorsosacral is the ancestral character state for all phytosaurs. Nonetheless, *Parasuchus hislopi*, a member of the non‐mystriosuchine parasuchid phytosaurs, was reported by Chatterjee (1978) to have only the two primordial sacral vertebrae. A reexamination of the two articulated skeletons of *P. hislopi* (ISI R 42 and ISI R 43) is required to determine whether a dorsosacral is truly absent in this species and to establish the polarity of this character within Phytosauria.

### 4.4 Identifying vertebral homologies in the sacra of other archosauriforms

Nesbitt (2011) presented criteria for identifying the homologies of the sacral vertebrae in archosauriforms with more than two sacral vertebrae. He regarded these criteria as a repeatable methodology, with the caveat that they had not been fully tested. We found that these criteria yielded consistent results in phytosaurs, allowing the identification of the primordial sacral vertebrae in all cases, including from specimens where only the medial portions of the ilia were preserved. We offer the caution that the primordial second sacral rib morphology must be examined closely, because the incorporation of a caudosacral vertebra into the sacrum may involve changes in the morphology of the primordial second sacral rib. In PEFO 31219, the facet on the primordial second sacral rib for the caudosacral rib gives the second sacral rib a more robust morphology in lateral view. Without considering other aspects of its morphology (e.g., the thin posterior shelf of the rib, the direction of dip in lateral view, etc.) and the placement of the rib scars on the medial ilium, it may have been misidentified as an insertion.

### 4.5 Paleobiological inferences

The addition of vertebrae to the sacrum likely adds support and stability to the sacro‐iliac joint, which is the only osteological linkage between the axial and appendicular skeletons. This would likely be advantageous to animals with a larger body size and/or to those that engage in more terrestrial locomotion. Sauropodomorph dinosaurs show a correlation between higher sacral numbers and larger body size (Moro et al., 2021; Sander et al., 2011), as do dicynodont therapsids (Griffin & Angielczyk, 2019). Scheyer et al. (2019) followed the body size interpretation for the presence of a dorsosacral vertebra in a specimen of the giant, morphologically divergent Miocene caimanine *Purussaurus mirandai* of South America, which they estimated as ranging between 7.11–8.01 m in length and 1686–2637 kg in mass. Unfortunately, the presence of a dorsosacral has not been confirmed in other giant extinct crocodylians (Scheyer et al., 2019). Phytosaurs could also attain enormous sizes, with the largest complete skull ever described (*Smilosuchus gregorii*, AMNH FARB 3060) being just over 1.4 m long (Colbert, 1947). One extremely large, partial “*Parasuchus*‐type” skull was estimated, when complete, as being just over 1.5 m long (Lucas et al., 2007), indicating that non‐mystriosuchine phytosaurs could attain sizes just as great as those of mystriosuchine leptosuchomorph phytosaurs. Furthermore, the recently described mystriosuchine non‐leptosuchomorph *Colosossuchus techniensis* (Datta & Ray, 2023) is estimated to have reached a total body length of more than 8 m, making it one of the largest phytosaurs known. PEFO 31219 is no exception, having a skull measuring over 1 m in length (Ballew, 1989). Given these facts, it is reasonable to speculate that additional vertebrae in the sacrum were advantageous for phytosaurs that attained large body sizes. Nevertheless, an additional sacral vertebra is also present in *Diandongosuchus fuyuanensis* (skull length = 0.24 m; Li et al. (2012)), which, if assumed to be the basalmost phytosaur, would indicate that the addition of a third sacral occurred early in the phytosaur lineage before attaining great size, or that it was acquired independently in *D. fuyuanensis* as well as in parasuchid phytosaurs. Alternatively, the presence of a third sacral may have enabled phytosaurs to attain such large body sizes rather than being a direct adaptation to large body size.

The addition of sacral vertebrae is also advantageous for terrestrial locomotion because it strengthens the articulation between the pelvic girdle and the axial column, allowing for better transmission of forces from the hindlimb to the rest of the body (LeBlanc & Reisz, 2015). Additional sacral vertebrae with sacral fusion is a feature seen in poposauroid pseudosuchian archosaurs (e.g., *Poposaurus*, *Effigia*; Schachner et al., 2020; Nesbitt, 2007) and small ornithischian (e.g., *Heterodontosaurus, Lesothosaurus*) and theropod (e.g., *Megapnosaurus*, *Coelophysis*) dinosaurs (Moro et al., 2021), which displayed an erect posture suited for efficient terrestrial locomotion, as well as bipedalism in certain taxa. Certain extinct crocodyliforms possess additional sacral vertebrae (e.g., the theropod‐like baurusuchids; Riff and Kellner (2011)), which may have supported their erect posture and terrestrial locomotion (Scheyer et al., 2019). Based on the apparently phytosaurian ichnogenus *Apatopus*, Padian et al. (2010) argued for the ability of phytosaurs to use the high walk (also used by extant crocodylians) wherein the femur was held close to the body in an erect posture when standing and a parasagittal gait when walking. Certain phytosaur taxa (e.g., *Nicrosaurus*) show an apparently greater secondary adaptation to a terrestrial lifestyle, possessing straighter, less sigmoid femora and dorsoventrally expanded ilia similar to those of fully terrestrial archosaurs (Kimmig, 2013). Skull morphology may also indicate a more terrestrial lifestyle in certain phytosaur taxa. The skull of PEFO 31219 is described by Holloway (2018) as brachyrostral (possessing a rostrum with an intermediate width‐length ratio) and having a rostral crest. Hunt (1989) interpreted phytosaurs with this skull morphology as being analogous to extant crocodylians that possess moderate‐length, massive rostra and engage in more terrestrial predation (e.g., *Crocodylus niloticus*). For this reason, we speculate that *M. mccauleyi* may have been predating on larger, more terrestrial prey and may have engaged in more terrestrial locomotion than *Machaeroprosopus* species with a dolichorostral (narrow, elongate) skull‐shape (e.g., *M. pristinus*; but see Holloway, 2018). The addition of a third or fourth sacral vertebra in PEFO 31219 may support this hypothesis, as a more stabilized pelvis‐axial column connection would be more supportive of a terrestrial lifestyle.

Could the sacral vertebral count differences in phytosaurs be related to sexual differences, as has been suggested for certain dinosaur taxa (Galton, 1999)? Sexual dimorphism in phytosaurs has been argued for on the basis on differences in the degree of elevation of the external nares above the skull roof (Camp, 1930; Colbert, 1947) and the degree of development of a rostral crest (Zeigler et al., 2002). Kimmig and Spielmann (2011) noted that in several Upper Triassic deposits, two morphs (with large and small crests) are observed, which may give support to this hypothesis. However, only one pair of phytosaur taxa posited as being different sexes of the same species appear as having a sister relationship in phylogenetic analyses (Jones & Butler, 2018a): *Machaeroprosopus buceros* (large crest, male) and *M. pristinus* (small crest, female), the skulls of which co‐occur in the Canjilon Quarry in New Mexico, U.S.A. (Zeigler et al., 2002). Unfortunately, the postcranial material for these individuals is not of sufficient quality or association with identifiable skulls to test whether sacral count correlates with sex (see discussion above regarding UCMP 34253).

The degree of fusion that occurs in the sacrum/synsacrum is known to vary ontogenetically in non‐avian dinosaurs (Moro et al., 2021) and in extant and extinct birds (Benito et al., 2022; Hogg, 1982) as well as in pterosaurs (Bennett, 2001; Dalla Vecchia, 2018). However, given that vertebral identity is determined in development, it seems unlikely that the addition of either a dorsosacral or caudosacral vertebra could be a consequence of changes in postnatal ontogeny.

## 5 CONCLUSION

Studies of phytosaur relationships and paleoecology have, thus far, focused mostly on cranial material. Though our picture of their relationships have been significantly modified over the past two decades, considerable ambiguity remains as to the relationships of some major groups of phytosaurs (e.g., Leptosuchomorpha). Though some studies have revealed examples of postcranial variation in phytosaurs, including some that is likely taxonomically and paleoecologically significant, more detailed descriptions of phytosaur postcranial material is needed to evaluate the full scope of postcranial variation in phytosaurs.

Sacral vertebral count was previously shown to vary from two (the plesiomorphic condition) to three (i.e., the addition of a dorsosacral) vertebrae in phytosaurs. This study adds to our knowledge of sacral count variation in phytosaurs, with a clear example of a caudosacral vertebra in *Machaeroprosopus mccauleyi*. It also shows that the presence of a dorsosacral vertebra is very widespread, perhaps ubiquitous, in the phytosaurs of western (and possibly eastern) North America. More descriptions of phytosaur sacra and ilia, and re‐examinations of some key taxa (e.g., *Parasuchus hislopi*, *Rutiodon carolinensis*), are needed to ascertain whether a dorsosacral vertebra is truly universal in phytosaurs, and whether a caudosacral is present in any other specimens or taxa. Discerning the distribution of sacral counts in phytosaur taxa will help to reveal whether such variation can be explained as an adaptation to large body size and/or a more‐or‐less terrestrial lifestyle, or as intraspecific, sexual, or ontogenetic variation. If certain sacral vertebra counts can be demonstrated to be taxon‐specific, then sacral count may prove to be useful for reconstructing the relationships of certain phytosaur taxa.

## AUTHOR CONTRIBUTIONS

Caleb N. LePore: Research design/concept formulation, data acquisition, description of specimens, and drafting of manuscript. Matthew A. McLain: Advised research design/concept formulation, aided data acquisition and interpretation, and gave critical feedback and approval on the draft manuscript.

## Supporting information


Table S1.

Table S2.

Table S3.

## Data Availability

The data that supports the findings of this study that are not already presented in the text and figures of this article (i.e., measurements) are available in the supplementary material of this article.
